# *Staphylococcus epidermidis* Sensitizes Perinatal Hypoxic-Ischemic Brain Injury in Male but Not Female Mice

**DOI:** 10.3389/fimmu.2020.00516

**Published:** 2020-04-21

**Authors:** Giacomo Gravina, Pernilla Svedin, Maryam Ardalan, Ofer Levy, C. Joakim Ek, Carina Mallard, Jacqueline C. Y. Lai

**Affiliations:** ^1^Institute of Neuroscience and Physiology, Sahlgrenska Academy, University of Gothenburg, Gothenburg, Sweden; ^2^Precision Vaccines Program, Boston Children's Hospital, Boston, MA, United States; ^3^Department of Pediatrics, Harvard Medical School, Boston, MA, United States; ^4^Broad Institute of MIT and Harvard, Cambridge, MA, United States

**Keywords:** *Staphylococcus epidermidis*, bacterial infection, sepsis, hypoxia-ischemia, neonatal mice, brain injury, complement activation

## Abstract

**Background:** *Staphylococcus epidermidis* is the most common nosocomial infection and the predominant pathogen in late-onset sepsis in preterm infants. Infection and inflammation are linked to neurological and developmental sequelae and bacterial infections increase the vulnerability of the brain to hypoxia-ischemia (HI). We thus tested the hypothesis that *S. epidermidis* exacerbates HI neuropathology in neonatal mice.

**Methods:** Male and female C57Bl/6 mice were injected intraperitoneally with sterile saline or 3.5 × 10^7^ colony-forming units of *S. epidermidis* on postnatal day (PND) 4 and then subjected to HI on PND5 (24 h after injection) or PND9 (5 d after injection) by left carotid artery ligation and exposure to 10% O_2_. White and gray matter injury was assessed on PND14-16. In an additional group of animals, the plasma, brain, and liver were collected on PND5 or PND9 after infection to evaluate cytokine and chemokine profiles, C5a levels and C5 signaling.

**Results:** HI induced 24 h after injection of *S. epidermidis* resulted in greater gray and white matter injury compared to saline injected controls in males, but not in females. Specifically, males demonstrated increased gray matter injury in the cortex and striatum, and white matter loss in the subcortical region, hippocampal fimbria and striatum. In contrast, there was no potentiation of brain injury when HI occurred 5 d after infection in either sex. In the plasma, *S. epidermidis*-injected mice demonstrated increased levels of pro- and anti-inflammatory cytokines and chemokines and a reduction of C5a at 24 h, but not 5 d after infection. Brain CCL2 levels were increased in both sexes 24 h after infection, but increased only in males at 5 d post infection.

**Conclusion:** Ongoing *S. epidermidis* infection combined with neonatal HI increases the vulnerability of the developing brain in male but not in female mice. These sex-dependent effects were to a large extent independent of expression of systemic cytokines or brain CCL2 expression. Overall, we provide new insights into how systemic *S. epidermidis* infection affects the developing brain and show that the time interval between infection and HI is a critical sensitizing factor in males.

## Introduction

Extreme prematurity is associated with increased mortality and morbidity (1). Despite improved survival rates of preterm infants over the years, preterm birth remains a major health problem, especially for infants experiencing sepsis (2). Due to invasive procedures and extensive use of medical devices, certain infections pose a special risk to preterm infants (3, 4). The coagulase-negative staphylococci *Staphylococcus epidermidis* forms biofilms on medical devices and is one of the most common nosocomial infections in preterm infants and has emerged as the predominant pathogen in late-onset sepsis (5, 6).

Clinical and experimental evidence link perinatal infection and inflammation to subsequent neurological and developmental sequelae (7). Sepsis can induce neuroinflammation resulting in activation of neurotoxic processes (8). There is increased risk of neurodevelopmental impairment in infants that experience sepsis and a meta-analysis demonstrated that coagulase-negative staphylococci sepsis in very low birth weight infants is associated with a higher incidence of cerebral palsy (9). Furthermore, infection in preterm infants is associated with a greater incidence of subsequent cardiorespiratory events, such as apnoea and hypoxemia (10), and it is recognized that neonatal encephalopathy is likely multifactorial where both maternal and neonatal infections can exacerbate hypoxic-ischemic (HI) brain injury (11).

We and others have demonstrated that synthetic compounds, such as Pam_3_CSK_4_, a Toll-like receptor (TLR) 2 agonist that mimics aspects of inflammation driven by Gram-positive bacteria, increases the vulnerability of the brain to subsequent HI in neonatal mice (12, 13). Recently we extended these findings to show that live *S. epidermidis* bacterial infection induced 14 h prior to HI also sensitizes the brain to increased injury (14). However, the time interval between infection and subsequent HI is known to be important in experimental studies (15). Thus, to test the hypothesis that *S. epidermidis* infection increases the vulnerability to HI mainly during an ongoing infection, we used our model of self-clearing systemic *S. epidermidis* infection in neonatal mice to investigate the effects of infection on HI injury over time.

The complement system is an important component of innate host defense, enhancing killing of pathogens, and clearance of microbes. The complement system is impaired in preterm infants and has been associated with preterm birth and susceptibility to neonatal sepsis (16). The complement component 5 (C5) protein cleaves into two protein fragments upon activation: C5a and C5b. C5a signaling through C5a receptors plays an important role in the development of sepsis (17). C5 has also been implicated in cerebral injury (18, 19) and C5a is elevated in CSF of preterm infants (20). We therefore also investigated the involvement of C5 signaling following *S. epidermidis* infection. We demonstrate that the time interval between *S. epidermidis* infection and HI is critical in sensitizing the brain to HI injury and that the effects are sex-dependent as they were evident only in male mice.

## Materials and Methods

### Animals

C57Bl/6J wild-type mice were purchased from Janvier Labs (Le Genest-Saint-Isle, France) and Charles River Laboratories (Sulzfeld, Germany) and were bred in the animal facility at the University of Gothenburg (Experimental Biomedicine, University of Gothenburg). Mice were housed with a normal 12-h light/dark cycle (lights on at 06:00) and *ad libidum* access to standard laboratory chow diet (B&K, Solna, Sweden) and drinking water in a temperature controlled environment (20–22°C). All animal experiments were approved by the Gothenburg Animal Ethical Committee (No 663/2017). Mice of both sexes were used. Sex was established by visual inspection. In each experimental group, mice were obtained from at least three different litters.

### Study Design

**Figure 1:**
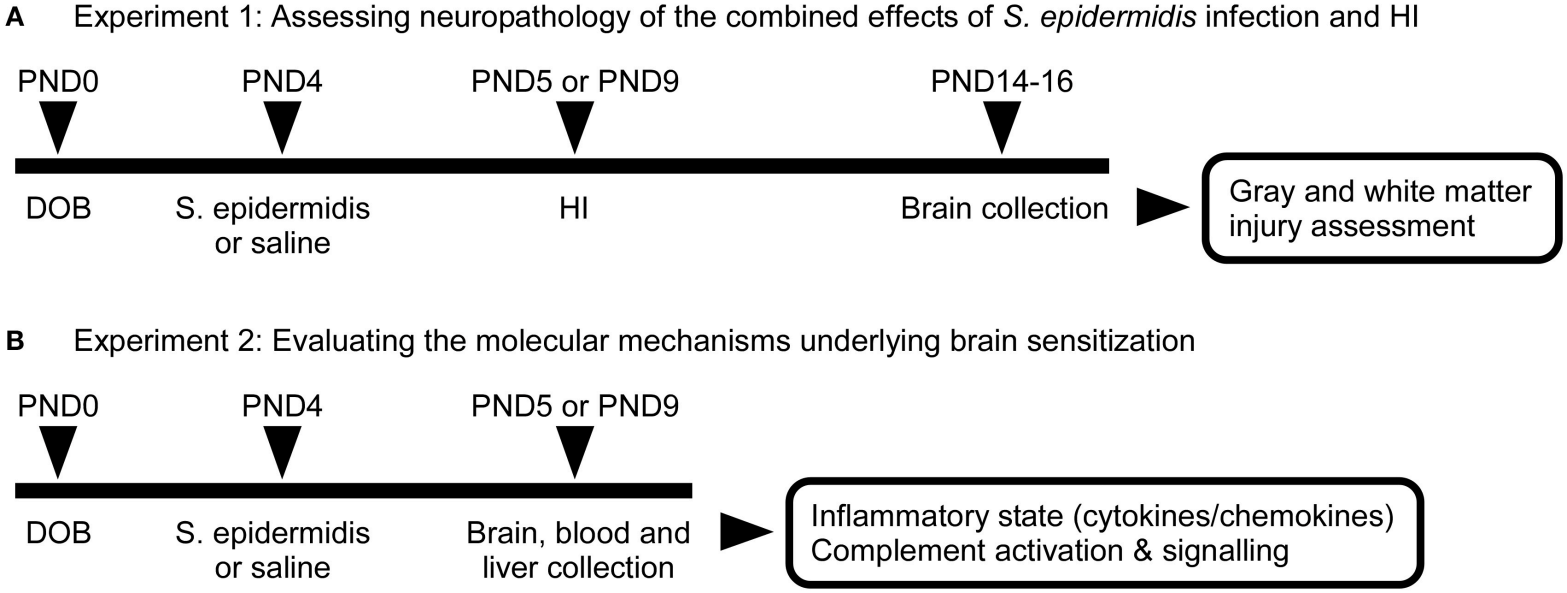
Schematic overview of experimental study design**. (A)** C57Bl/6J mice were injected intraperitoneally at PND4 with 3.5 × 10^7^ CFU of *S. epidermidis* or saline followed by hypoxia-ischemia (HI) procedure at 24 h (PND5) or 5 days (PND9) after injection. Brains were collected on PND14 or 16 for evaluation of neuropathology. **(B)** A second group of animals was sacrificed prior to HI for the evaluation of inflammatory response and complement activation and signaling in blood, liver, and brain.

We have previously shown that *S epidermidis* infection can increase the vulnerability of the developing brain to HI (14). To evaluate the potentiation of brain injury after *S. epidermidis* infection over time, mice were subjected to a combination of *S. epidermidis* infection and HI. For exposure to neonatal infection, mice were intraperitoneally injected with sterile saline or 3.5 × 10^7^ colony-forming units (CFU) of *S. epidermidis* at postnatal day (PND) 4 as previously described (14). HI was induced at 24 h (PND5) or 5 d (PND9) after *S. epidermidis* injection. The timing of HI was based on our previous study which showed that infection was ongoing at 24 h but largely cleared at 5 d (14). Animals were sacrificed on PND14-PND16 for neuropathological examination (Figure 1A).

To understand molecular mechanisms underlying brain sensitization at the time of HI, another group of animals was injected intraperitoneally at PND4 with 3.5 × 10^7^ CFU of *S. epidermidis* or saline (without HI). Twenty-four hours or 5 d after *S. epidermidis*/saline injection, blood, liver, and brain samples were collected for biochemical analysis (Figure 1B).

### Hypoxia-Ischemia Procedure

PND5 and PND9 pups were exposed to neonatal HI as previously described (21, 22). Pups were anesthetized with isoflurane (IsoFlo vet 100%; Abbott Laboratories Ltd, Illinois, USA), 5% for induction and 1.5% for maintenance, in 1:1 oxygen-nitrogen gas mixture. The left common carotid artery was ligated with a 7.0 silk suture (Ethicon; Vömel, Germany) and the incision was closed and infiltrated with a local anesthetic (Xylocain 20 mg/ml, lidocaine hydrochloride; Astra Zeneca, Södertälje, Sweden). After surgery, mice were returned to their dams for 1 h, before being placed in a chamber with circulating humidified air (36 °C). In the chamber, PND5 mice were exposed to 10 min of air, followed by 60 min of hypoxia (10% O_2_ in 90% N_2_) followed by another 10 min of air. PND9 mice were treated in the same manner except the hypoxia time was 50 min. This procedure results in a similar degree of brain injury in PND5 and PND9 mice as previously shown (21, 23) and Supplementary Figure 1. Following the hypoxic exposure, pups were returned to their dams until sacrifice.

### Tissue Preparation and Immunohistochemistry

PND14 (i.e., 9 d after HI at PND5) or PND16 (i.e., 7 d after HI at PND9) mice were deeply anesthetized via intraperitoneal administration of pentobarbital (Pentacour) and intracardially perfused with 0.9% saline followed by 6% buffered formaldehyde (Histofix; Histolab). The brains were collected and kept in the same fixative solution at 4°C until dehydration and paraffin embedding. For the immunohistochemistry analysis, brains were sectioned at 7-μm coronal thickness on a microtome and based on a systematic sampling principle every 50th section was used for immunohistochemical staining. Brain sections were heated at 65°C for 30 min, followed by deparaffinization in xylene and graded alcohol. Antigen retrieval was performed by boiling the tissue sections in 0.01 M citric acid buffer (pH 6.0). Brain sections were then washed in PBS, blocked for endogenous peroxidase activity with 3% H_2_O_2_ in PBS, followed by blocking for non-specific binding with 4% goat serum in PBS. Sections were incubated at 4°C overnight with primary antibody against microtubule-associated protein-2 (MAP-2; clone HM-2, 1:1,000; Sigma-Aldrich catalog # M4403) or myelin basic protein (MBP; clone SMI-94, 1:1,000; BioLegend catalog # 836504), followed by 1 h of incubation with horse-anti-mouse biotinylated secondary antibody (1:250; Vector Laboratories catalog # BA-2001) and VECTASTAIN Elite ABC HRP Kit (Vector Laboratories) according to manufacturer's instructions. Sections were visualized with a solution of 0.5 mg/ml 3,3-diaminobenzidine enhanced with 15 mg/ml ammonium nickel sulfate, 2 mg/ml β-D-glucose, 0.4 mg/ml ammonium chloride, and 0.01 mg/ml β-glucose oxidase (all from Sigma-Aldrich). In the last step, stained sections were dehydrated through a graded series of alcohol (70, 95, 99%), cleared in xylene and cover-slipped.

### Brain Injury Analysis

Gray matter injury was quantified on sections stained for MAP-2, which labels neurons and dendrites, and white matter was quantified on sections stained for MBP, which labels myelin. Images were captured on a light microscope (Olympus BX60) using a 4X objective lens. The region of interest (ROI) with MAP-2 or MBP positive immunoreactivity in the hemispheres ipsilateral and contralateral to the ligated artery were outlined and measured with ImageJ software (v1.52a, NIH, USA). Gray matter analysis was performed in brain regions ranging from the anterior striatum to the middle of the hippocampal structure (5 levels including the cortex, 3 levels including the dorsal hippocampus and thalamus, and 3 levels including the striatum). The sum of the MAP-2 positive areas in the cortex, hippocampus, thalamus, and striatum was considered as a measurement of the whole cerebral hemisphere.

Myelinated areas were determined as integrated density of MBP-positive staining (i.e., the product of area and mean gray value in the ROI). MBP staining was measured at 2 levels in the subcortical white matter, at 2 levels of the hippocampal fimbria, and at 3 levels in the striatum. The percentage of MAP-2 and MBP-positive tissue loss were calculated at each level as follows: [(contralateral side – ipsilateral side)/contralateral side × 100%]. The mean of the percentage tissue loss for all levels was compared between animals.

### Sample Collection for Biochemical Analysis

At 24 h (PND5) or 5 d (PND9) post infection or injection of saline (*n* = 10/treatment group/sex), mice were deeply anesthetized via intraperitoneal administration of pentobarbital (Pentacour). Approximately 20 μl of blood was collected via cardiac puncture and mixed with 5 μl of 50 mM EDTA. Mice were then transcardially perfused with 0.9% saline and brain and liver were collected and flash frozen in dry ice.

For protein analysis, brain and liver were homogenized in 1,000 μl of RNase-free PBS containing 0.5% protease inhibitor cocktail (Sigma catalog # P8340), 5 mM EDTA and 1% Triton X-100 by sonication (40% amplitude, 10 pulses, 1.2 s each). Lysates were then centrifuged (10,000 x g, 10 min, 4°C) and cleared supernatants were stored at −80°C. Protein concentrations were measured using the Pierce BCA Protein Assay Kit (Thermo Fisher Scientific) according to the manufacturer's instructions.

### Cytokine and Chemokine Assay

Bio-Plex Pro Mouse Cytokine Standard 23-Plex kit (Bio-Rad, catalog # M600009RDPD), analyzed on a Bio-Plex 200 System (Bio-Rad), was used to perform cytokine profiling of plasma from PND5 and PND9 mice according to the manufacturer's instructions. Plasma samples were diluted 1/5 in Bio-plex sample diluent prior to assaying.

### Measurement of Brain CCL2

Protein concentration of CCL2 from brain lysates was analyzed by enzyme-linked immunosorbent assay (ELISA) kit and standard (CCL2/JE/MCP-1 DuoSet ELISA kit, R&D Systems, catalog # DY479-05) as per manufacturer's instructions.

### Measurement of Plasma, Brain, and Liver C5a

C5a concentrations were measured using the mouse C5a DuoSet ELISA kit (R&D Systems, catalog # DY2150) as per manufacturer's instructions. Plasma from both PND5 and PND9 mice were diluted 1/200, whereas brain lysates were diluted 1/50 in reagent diluent. Liver lysates from PND5 and PND9 samples were diluted 1/50 and 1/200 respectively.

### Measurement of C5, C5aR1, and C5aR2 mRNA Expression in Brain and Liver

Total RNA was prepared from the brain and liver lysates in RNase-free PBS using RNeasy Mini Kit (Qiagen), following the manufacturer's instructions and measured using NanoDrop 2000 (Thermo Fisher Scientific). RNA was reversed transcribed into cDNA using QuantiTect Reverse Transcription Kit (Qiagen). All cDNA samples were diluted with nuclease-free water to a final volume of 50 μl. Quantitative real-time RT-qPCR was performed with a Touch real-time cycler (Bio-Rad, Hercules, CA, USA). Each 20 μl reaction contained 10 μl Fast SYBR master mix (Qiagen), 2 μl of 10x Primer set (Qiagen) for C5 - QT00102032; C5a receptor 1 (C5aR1) - QT00288232 and C5a receptor 2 (C5aR2) - QT02532803, 6 μl of H_2_O and 2 μl of cDNA. The PCR temperature profile was 95 °C for 2 min followed by 40 cycles of amplification (95 °C for 10 s and 60 °C for 30 s).

The values for each gene were normalized to the concentration of cDNA in each RT sample measured using the QUANT-IT™ OLIGREEN ssDNA assay kit (Invitrogen), according to the manufacturer's instructions. The amount of target gene expression was calculated as follows: ([cDNA_targetgene_/average of cDNA_total_ in all samples]/total cDNA_targetgene_).

### Statistical Analysis

Statistical analyses were performed using GraphPad Prism v.8. Descriptive data are presented as box plots with median and the whiskers at 10–90th percentile. Normal distribution of the data was examined by generating Q-Q plot and in cases of non-normal distribution, log transformation of data was performed. The interaction between the independent variables (bacterial infection and sex) and the main effect of sex or bacterial infection on brain injury were tested using two-way analysis of variance (Two-way ANOVA). *Post-hoc* analysis between the groups was performed using the Sidak's multiple comparison test. *P*-values < 0.05 were considered statistically significant.

## Results

### *Staphylococcus epidermidis* Sensitizes Hypoxic-Ischemic Brain Injury in Males but Not in Females

To assess the window of increased vulnerability to HI after *S. epidermidis* bacteraemia, we induced HI either at 24 h post infection, when bacteraemia was high, or at 5 d after infection, when bacteria was significantly reduced in the blood (14). We assessed changes in the white and gray matter at PND14—PND16, corresponding to an age when myelination is established and detectable by immunohistochemical staining in mice (24). As infection and inflammation can affect perinatal brain injury in a sex-dependent manner, reviewed in Ardalan et al. (25), neuropathological outcome was determined in male and female pups separately.

**Figure 2:**
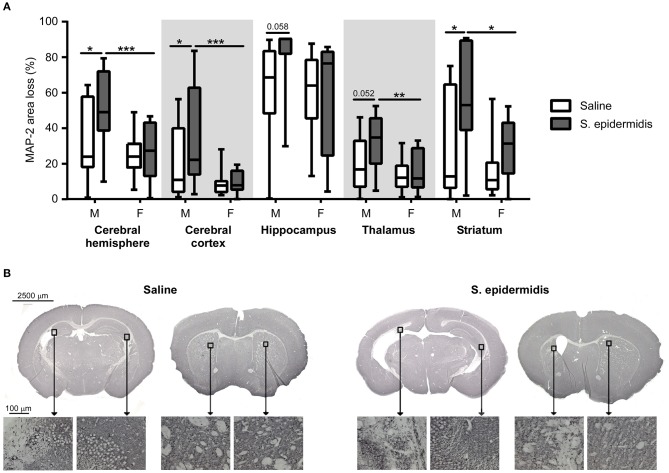
*S. epidermidis* 24 h prior to hypoxia-ischemia potentiates gray matter injury in neonatal male mice. PND4 mice were injected with saline or *S. epidermidis* and subjected to hypoxia-ischemia 24 h later. **(A)** Gray matter brain injury was assessed by microtubule-associated protein 2 (MAP-2) immunohistochemistry at PND14 in the entire cerebral hemisphere, cerebral cortex, hippocampus, thalamus, and striatum (*n* = 12 saline males; *n* = 11 *S. epidermidis* males; *n* = 17 saline females, and *n* = 13 *S. epidermidis* females). **(B)** Representative images (4 × objective lens) of PND14 male mice brain sections stained with MAP-2 at the hippocampal and striatal levels following saline or *S. epidermidis* injection at PND4 in combination with hypoxia-ischemia 24 h later. Images of CA3 region of the hippocampus and striatum captured on a light microscope with a 20× objective lens. Data are presented as median and 10–90^th^ percentile. Statistical comparison between the *S. epidermidis* and saline groups for each brain region was performed using Two-way ANOVA with Sidak's multiple comparison *post-hoc* test; *p < 0.05, **p < 0.01, and ***p < 0.001.

*S. epidermidis* bacteraemia initiated 24 h before HI potentiated brain injury in male but not in female mice (Figure 2). Two-way ANOVA of regional gray matter analysis revealed a significant interaction between sex and bacterial infection in the cortex [*F*_(1, 48)_ = 4.28, *p* = 0.04]. Significant main effects of bacterial infection were seen in the striatum and total hemispheres [*F*_(1, 48)_ = 8.08, *p* = 0.006; *F*_(1, 48)_ = 4.68, *p* = 0.03], while significant main effects of sex were observed in the cortex, thalamus, striatum, and total hemispheres respectively [*F*_(1, 48)_ = 12.13, *p* = 0.001; *F*_(1, 48)_ = 8.03, *p* = 0.006; *F*_(1, 48)_ = 6.47, *p* = 0.01; *F*_(1, 48)_ = 7.81, *p* = 0.006]. Tissue loss in the entire cerebral hemisphere was greater in *S. epidermidis* infected male mice compared to saline treated males (*p* = 0.01), but not in female mice. Specifically, regional gray matter analysis revealed that *S. epidermidis* infected male mice had increased tissue loss in the cerebral cortex (*p* = 0.02) and in the striatum (*p* = 0.01) compared to saline treated male mice. Significantly increased injury in infected male mice compared to female mice was found when analyzing the entire hemisphere (*p* = 0.004), cortex (*p* = 0.0008), thalamus (*p* = 0.005) and striatum (*p* = 0.01). *S. epidermidis* did not increase gray matter tissue loss in female mice compared to saline-injected controls in any region examined.

**Figure 3:**
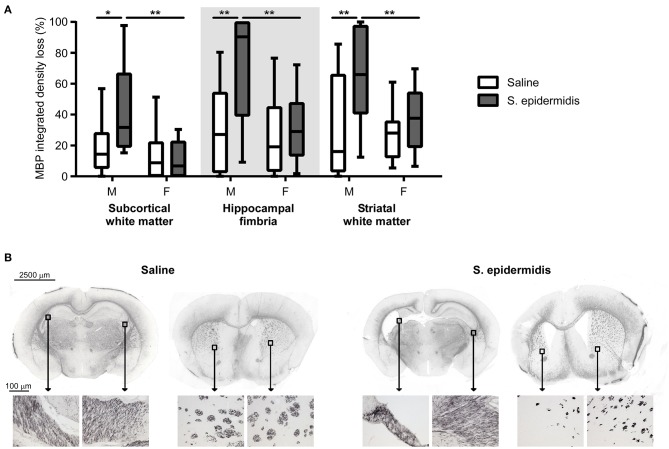
*S. epidermidis* 24 h prior to hypoxia-ischemia potentiates white matter injury in neonatal male mice. PND4 mice were injected with saline or *S. epidermidis* and subjected to hypoxia-ischemia 24 h later. **(A)** White matter brain injury was assessed by myelin basic protein (MBP) immunohistochemistry at PND14 in the subcortical region, hippocampal fimbria, and striatum of male (M) and female (F) mice (*n* = 12 saline males; *n* = 11 *S. epidermidis* males; *n* = 16 saline females, and *n* = 13 *S. epidermidis* females). **(B)** Representative images (4 × objective lens) of PND14 male mice brain sections stained with MBP at the hippocampal and striatal levels following saline or *S. epidermidis* injection at PND4 in combination with hypoxia-ischemia 24 h later. Images of the hippocampal fimbria and striatum captured on a light microscope with a 20× objective lens. Data are presented as median and 10–90^th^ percentile. Statistical comparison between the *S. epidermidis* and saline groups for each brain region was performed using Two-way ANOVA with Sidak's multiple comparison *post-hoc* test; *p < 0.05 and **p < 0.01.

*S. epidermidis* bacteraemia initiated 24 h before HI potentiated white matter injury in male but not female mice (Figure 3). There was a significant interaction between sex and bacterial infection and main effect of sex on white matter injury in the subcortical region [*F*_(1, 47)_ = 4.82, *p* = 0.01; *F*_(1, 47)_ = 7.92, *p* = 0.007] and hippocampal fimbria [*F*_(1, 47)_ = 4.82, *p* = 0.03; *F*_(1, 47)_ = 7.58, *p* = 0.008]. Additionally, an effect of bacterial infection on white matter loss was detected in the fimbria [*F*_(1, 47)_ = 8.36, *p* = 0.005] and striatum [*F*_(1, 47)_ = 8.48, *p* = 0.005]. *Post-hoc* analysis demonstrated an increased white matter tissue loss in *S. epidermidis* infected male mice compared with saline controls in the subcortical white matter (*p* = 0.01), hippocampal fimbria (*p* = 0.003), and striatum (*p* = 0.004). Consistent with MAP-2 results, a significant increase in tissue loss was found in the subcortical white matter (*p* = 0.001), hippocampal fimbria (*p* = 0.003), and striatum (*p* = 0.02) in *S. epidermidis* infected male mice compared to *S. epidermidis* infected female mice. *S. epidermidis* did not increase white matter tissue loss in female mice compared to saline-injected controls in any region examined.

**Figure 4:**
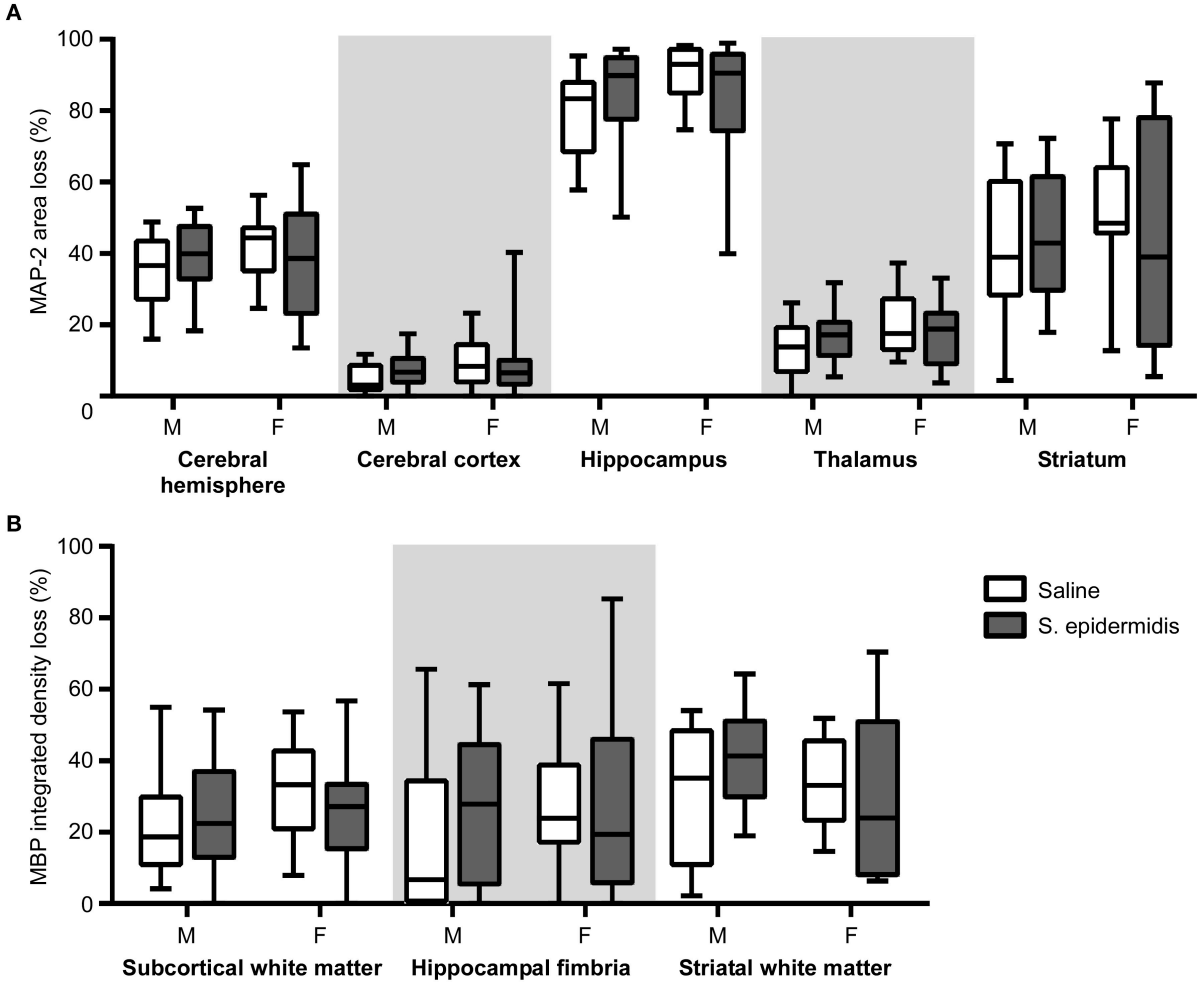
*S. epidermidis* 5 days prior to hypoxia-ischemia does not potentiate neonatal brain injury. PND4 mice were injected with saline or *S. epidermidis* and subjected to hypoxia-ischemia 5 days later. **(A)** Gray matter brain injury was assessed by MAP-2 immunohistochemistry at PND16 in the entire cerebral hemisphere, cerebral cortex, hippocampus, thalamus, and striatum of male (M) and female (F) mice. **(B)** White matter brain injury was assessed by MBP immunohistochemistry at PND16 in the subcortical region, hippocampal fimbria, and striatum. *n* = 12 saline males; *n* = 15 *S. epidermidis* males; *n* = 16 saline females, and *n* = 14 *S. epidermidis* females. Data are presented as median and 10–90^th^ percentile. Statistical comparison was performed between the *S. epidermidis* and saline groups for each brain region using Two-way ANOVA with Sidak's multiple comparison *post-hoc* test.

When the time interval between *S. epidermidis* infection and HI was extended to 5 days, there was no significant interaction between sex and bacterial infection, and no significant effects of sex and bacterial infection on the degree of gray or white matter tissue loss (Figure 4). No significant differences in baseline HI injury were seen between male and female mice at either age after saline and HI treatment (Figures 2–4). Similarly, there was no difference in overall hemispheric injury between animals subjected to saline and HI at PND5 and PND9 (Supplementary Figure 1).

### *S. epidermidis* Induced Cytokine and Chemokine Production in Blood

**Figure 5:**
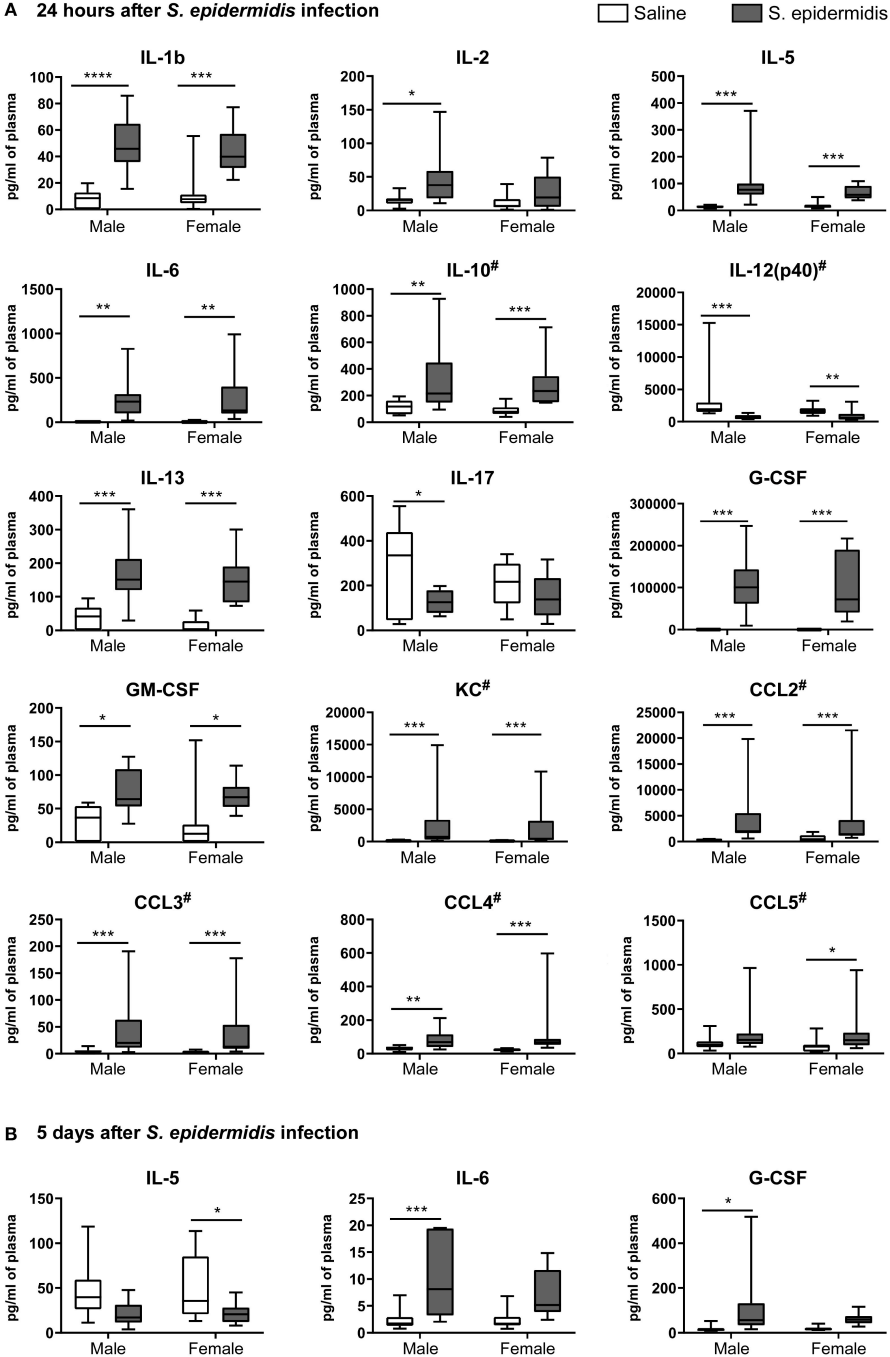
*S. epidermidis* infection induces plasma pro-inflammatory cytokines and chemokines. PND4 mice were injected with saline or *S. epidermidis* and the plasma analyzed for cytokines and chemokines at **(A)** 24 h or **(B)** 5 days (*n* = 10 mice/sex/group). Data are presented as median and 10–90^th^ percentile. Statistical comparison between the *S. epidermidis* and saline groups was performed using Two-way ANOVA with Sidak's multiple comparison *post-hoc* test; *p < 0.05, **p < 0.01, and ***p < 0.001. # indicates data presented before log transformation.

To evaluate inflammatory processes associated with increased vulnerability to HI injury, we examined animals 24 h and 5 d after *S. epidermidis* or saline injection, prior to HI. We first measured inflammatory cytokine concentrations in the plasma using a 23-plex cytometry bead array. Two-way ANOVA of plasma cytokine concentrations revealed no significant interaction between sex and bacterial infection at 24 h or 5 d after infection (Tables 1, 2), but there was a significant main effect of bacterial infection on the plasma cytokine and chemokines levels at 24 h, including IL-1b [*F*_(1, 36)_ = 48.45, *p* < 0.0001], IL-2 [*F*_(1, 36)_ = 16.53, *p* = 0.0002], IL-5 [*F*_(1, 36)_ = 21.71, *p* < 0.0001], IL-6 [*F*_(1, 36)_ = 19.32, *p* < 0.0001], IL-10 [*F*_(1, 36)_ = 31.16, *p* < 0.0001], IL-12(p40) [*F*_(1, 36)_ = 33.7, *p* < 0.0001], IL-13 [*F*_(1, 36)_ = 44.09, *p* < 0.0001], IL-17 [*F*_(1, 36)_ = 6.99, *p* = 0.012], G-CSF [*F*_(1, 36)_ = 41.28, *p* < 0.0001], GM-CSF [*F*_(1, 36)_ = 16.36, *p* = 0.0003], KC [*F*_(1, 36)_ = 34.86, *p* < 0.0001], CCL2 (MCP-1) [*F*_(1, 36)_ = 45.44, *p* < 0.0001], CCL3 (MIP1a) [*F*_(1, 36)_ = 34.57, *p* < 0.0001], CCL4 (MIP1b) [*F*_(1, 36)_=30.77, *p* < 0.0001], and CCL5 (RANTES) [*F*_(1, 36)_ = 10.51, *p* = 0.002] (Figure 5A). The levels of IL-2, IL-17 and CCL5 at 24 h after infection were regulated in a sex-dependent manner. Specifically, we observed an upregulation of IL-2 (*p* = 0.02) in males and CCL5 (*p* = 0.01) in females, while IL-17 was downregulated in males (*p* = 0.02) (Figure 5A, Table 1). At 5 days after infection, IL-6 and G-CSF were increased in males, whereas IL-5 and G-CSF were reduced in *S. epidermidis* infected females compared to control animals (Figure 5B, Table 2).

**Table 1:** Cytokines and chemokines 24 h after *S. epidermidis* infection.

**Treatment**	**Saline**		***S. epidermidis***		***p*-value^a^^,^^b^^,^^c^**
**Sex**	**Male**	**Female**	**Male**	**Female**	
**Cytokines**					
IL-1a	138.6 ± 221.5	73.2 ± 179.8	112.4 ± 43.3	102.6 ± 64.8	n.s.
IL-1b	7.6 ± 7.4	12.1 ± 17.2	48.4 ± 21.0	44.7 ± 17.7	***; ###
IL-2	15.2 ± 8.6	12.1 ± 12.2	47 ± 42.4	28.5 ± 27.2	*
IL-3	15.3 ± 11.3	12.5 ± 8.3	16.6 ± 5.8	17.4 ± 6.5	n.s.
IL-4	11.3 ± 21.2	0.4 ± 0.5	5.1 ± 10.6	5.2 ± 10.3	n.s.
IL-5	13.8 ± 5.1	18.2 ± 13.3	104.4 ± 106.7	66.7 ± 25.2	***; ###
IL-6	3.5 ± 2.2	4.7 ± 8.3	267.2 ± 239.7	285.9 ± 310.1	**; ##
IL-9	26.8 ± 17.4	19.4 ± 14.9	19.5 ± 12.0	19.7 ± 11.5	n.s.
IL-10	113.6 ± 54.5	89.3 ± 41.9	328 ± 280.7	283.9 ± 187.0	**; ###
IL-12 (p40)	3511 ± 4642	1769 ± 713.8	701.0 ± 354.4	895.9 ± 906.7	***; ##
IL-12 (p70)	344.3 ± 230.0	181.7 ± 140.7	194.0 ± 158.7	199.7 ± 140.6	n.s.
IL-13	41.1 ± 36.0	14.4 ± 22.2	169.1 ± 95.4	153.4 ± 72.5	***; ###
IL-17	280.3 ± 202.3	206.2 ± 104.6	127.9 ± 49.3	148.2 ± 95.0	*
Eotaxin	1082 ± 297.5	882.1 ± 300.5	1069 ± 454.3	1000 ± 423.7	n.s.
G-CSF	55.9 ± 69.9	38.6 ± 45.3	108582 ± 70384	104658 ± 77792	***; ###
GM-CSF	28.8 ± 25.6	26.9 ± 50.1	74.3 ± 33.6	70.4 ± 23.5	*; #
IFN-g	40.9 ± 27.4	25.2 ± 15.9	29.8 ± 14.4	29.7 ± 16.1	n.s.
KC	121.7 ± 75.4	96.4 ± 61.2	2735 ± 4909	2237 ± 3567	***; ###
CCL2	318.1 ± 143.2	671.9 ± 606.1	4581 ± 6254	4284 ± 6872	***; ###
CCL3	4.8 ± 4.1	3.0 ± 2.0	45.2 ± 61.6	41.1 ± 56.6	***; ###
CCL4	31.8 ± 13.4	23.1 ± 7.5	85.1 ± 62.5	124.6 ± 186.3	**; ###
CCL5	117.9 ± 81.6	86.5 ± 82.8	248.1 ± 285.7	236.1 ± 281.7	#
TNF-a	126.9 ± 88.3	99.2 ± 54.8	141.4 ± 57.4	136.0 ± 44.7	#

*Postnatal day 4 mice were intraperitoneally injected with saline or 3.5 × 10^7^ CFU of S. epidermidis. Plasma samples were obtained 24 h later for cytometric bead array analysis*.

a Saline vs. S. epidermidis-injected male mice *p < 0.05, **p < 0.01, and ***p < 0.001.

b *Saline vs. S. epidermidis-injected female mice ^#^p < 0.05, ^##^p < 0.01, and ^###^p < 0.001*.

c *No statistical differences were observed between male and female mice injected with saline, or between male and female mice injected with S. epidermidis*.

**Table 2:** Cytokines and chemokines 5 days after *S. epidermidis* infection.

**Treatment**	**Saline**		***S. epidermidis***		***p*-value^a^^,^^b^^,^^c^**
**Sex**	**Male**	**Female**	**Male**	**Female**	
**Cytokines**					
IL-1a	48.2 ± 55.5	47.3 ± 51.1	10.5 ± 5.6	21.8 ± 19.8	n.s.
IL-1b	7.3 ± 8.2	3.3 ± 5.7	8.7 ± 8.5	7.5 ± 11.3	n.s.
IL-2	10.0 ± 5.4	12 ± 9.3	8.6 ± 4.9	13.2 ± 11.1	n.s.
IL-3	11.1 ± 4.9	11.7 ± 9.8	10.6 ± 6.5	12.5 ± 8.4	n.s.
IL-4	0.2 ± 0.0	2.4 ± 6.9	0.4 ± 0.7	1.5 ± 4.1	n.s.
IL-5	46.6 ± 32.4	51 ± 37.3	20.8 ± 13.7	21.7 ± 11.8	#
IL-6	2.4 ± 1.9	2.4 ± 1.9	10.3 ± 7.3	6.9 ± 4.3	***
IL-9	16.1 ± 11.8	17.0 ± 15.9	13.6 ± 9.4	14.7 ± 13.0	n.s.
IL-10	102.0 ± 87.4	74.9 ± 36.5	111.6 ± 45.7	110.3 ± 49.1	n.s.
IL-12 (p40)	1294 ± 304.6	1316 ± 390.8	1221 ± 536.1	1036 ± 463.8	n.s.
IL-12 (p70)	212.5 ± 156.6	213.3 ± 229.6	131.9 ± 115.1	186.4 ± 198.4	n.s.
IL-13	3.164 ± 6.2	7.6 ± 22.8	4.5 ± 12.9	9.9 ± 30.2	n.s.
IL-17	290.9 ± 83.5	276.6 ± 202.0	187.5 ± 142.5	259.8 ± 147.2	n.s.
Eotaxin	806.0 ± 254.4	877.3 ± 278.3	574.4 ± 190.3	617 ± 180.3	n.s.
G-CSF	18.0 ± 13.6	18.3 ± 9.0	115.6 ± 161.8	62.3 ± 27.7	*
GM-CSF	7.7 ± 13.0	14.3 ± 20.9	15.1 ± 20.2	12.6 ± 19.0	n.s.
IFN-g	28.3 ± 16.4	25.3 ± 25.5	18.8 ± 15.9	23.7 ± 19.1	n.s.
KC	71.2 ± 31.4	86.9 ± 46.2	98.9 ± 36.1	90.2 ± 30.4	n.s.
CCL2	874.2 ± 989.7	271.2 ± 396.5	529.1 ± 750.0	783 ± 1017	n.s.
CCL3	2.6 ± 1.3	2.7 ± 2.0	3.5 ± 1.6	3.8 ± 1.6	n.s.
CCL4	21.8 ± 8.6	22.6 ± 11.9	27.3 ± 9.9	28.9 ± 11.5	n.s.
CCL5	61.2 ± 22.7	61.2 ± 29.7	109.3 ± 63.5	121.8 ± 113.1	n.s.
TNF-a	94.8 ± 48.1	99.6 ± 81.2	79.6 ± 50.8	96.5 ± 63.5	n.s.

*Postnatal day 4 mice were intraperitoneally injected with saline or 3.5 × 10^7^ CFU of S. epidermidis. Plasma samples were obtained 5 days later for cytometric bead array analysis*.

a Saline vs. S. epidermidis-injected male mice *p < 0.05, and ***p < 0.001.

b *Saline vs. S. epidermidis-injected female mice ^#^p < 0.05*.

c *No statistical differences were observed between male and female mice injected with saline, or between male and female mice injected with S. epidermidis*.

**Figure 6:**
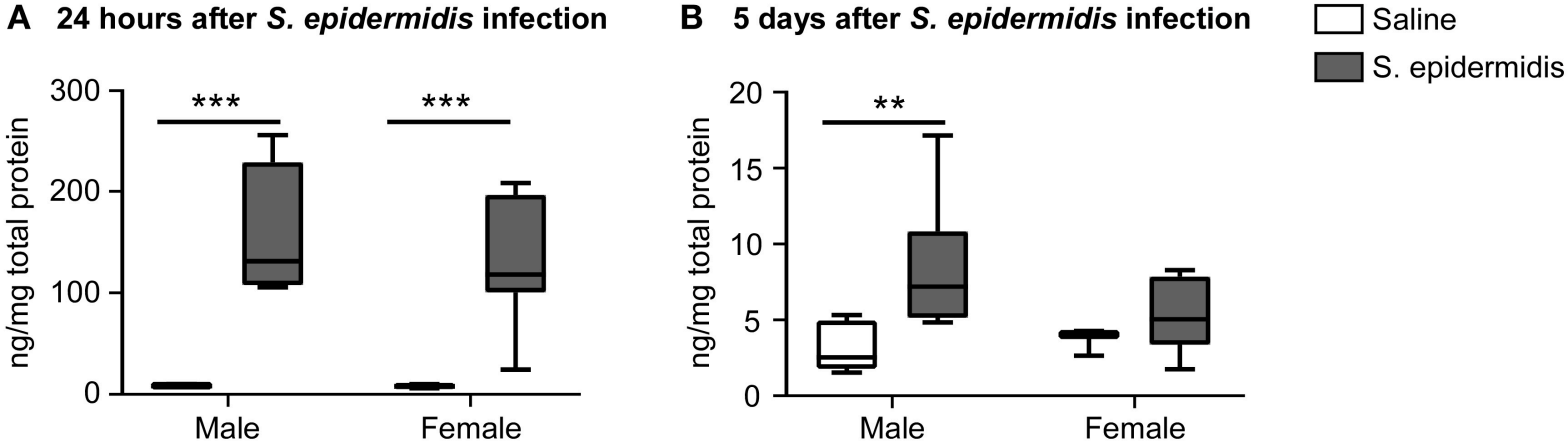
CCL2 levels are increased in the brain following *S. epidermidis* infection. PND4 mice were injected with saline or *S. epidermidis* and analyzed at **(A)** 24 h (*n* = 5 saline males; *n* = 4 *S. epidermidis* males; *n* = 4 saline females, and *n* = 8 *S. epidermidis* females) or **(B)** 5 days (*n* = 7 saline males; *n* = 6 *S. epidermidis* females; *n* = 3 saline females, and *n* = 7 *S. epidermidis* females) after infection. CCL2 brain concentrations are presented as median and 10–90^th^ percentile. Statistical comparison between the *S. epidermidis* and saline groups was done using Two-way ANOVA followed by Sidak's multiple comparison *post-hoc* test; **p < 0.01 and ***p < 0.001.

To assess whether *S. epidermidis* induces an inflammatory response in the immature brain, concentrations of CCL2 in brain homogenates 24 h and 5 d after infection were evaluated by ELISA (Figure 6). CCL2 levels in the brain ranged from 6.2 to 255 μg/ml at 24 h (Figure 6A), but from 1.5 to 17.1 μg/ml at 5 d (Figure 6B) after infection. Analysis of brain CCL2 concentrations revealed no significant interaction between sex and bacterial infection at 24 h or 5 days after infection, but there was a significant effect of bacterial infection on brain CCL2 levels at 24 h [*F*_(1, 17)_ = 36.84, *p* < 0.0001] and 5 d [*F*_(1, 19)_ = 7.20, *p* = 0.01]. Compared to their respective saline cohorts, brain CCL2 concentrations in infected mice at 24 h were significantly higher in both males and females (18.2-fold in males, *p* = 0.007; 16.4-fold in females, *p* = 0.001) (Figure 6A). At 5 days post infection, there was still an increase in CCL2 in infected males (2.1-fold, *p* = 0.006), although less augmented than at 24 h (Figure 6B).

### *S. epidermidis* Bacteraemia Affects Complement Component C5a Release in the Plasma

**Figure 7:**
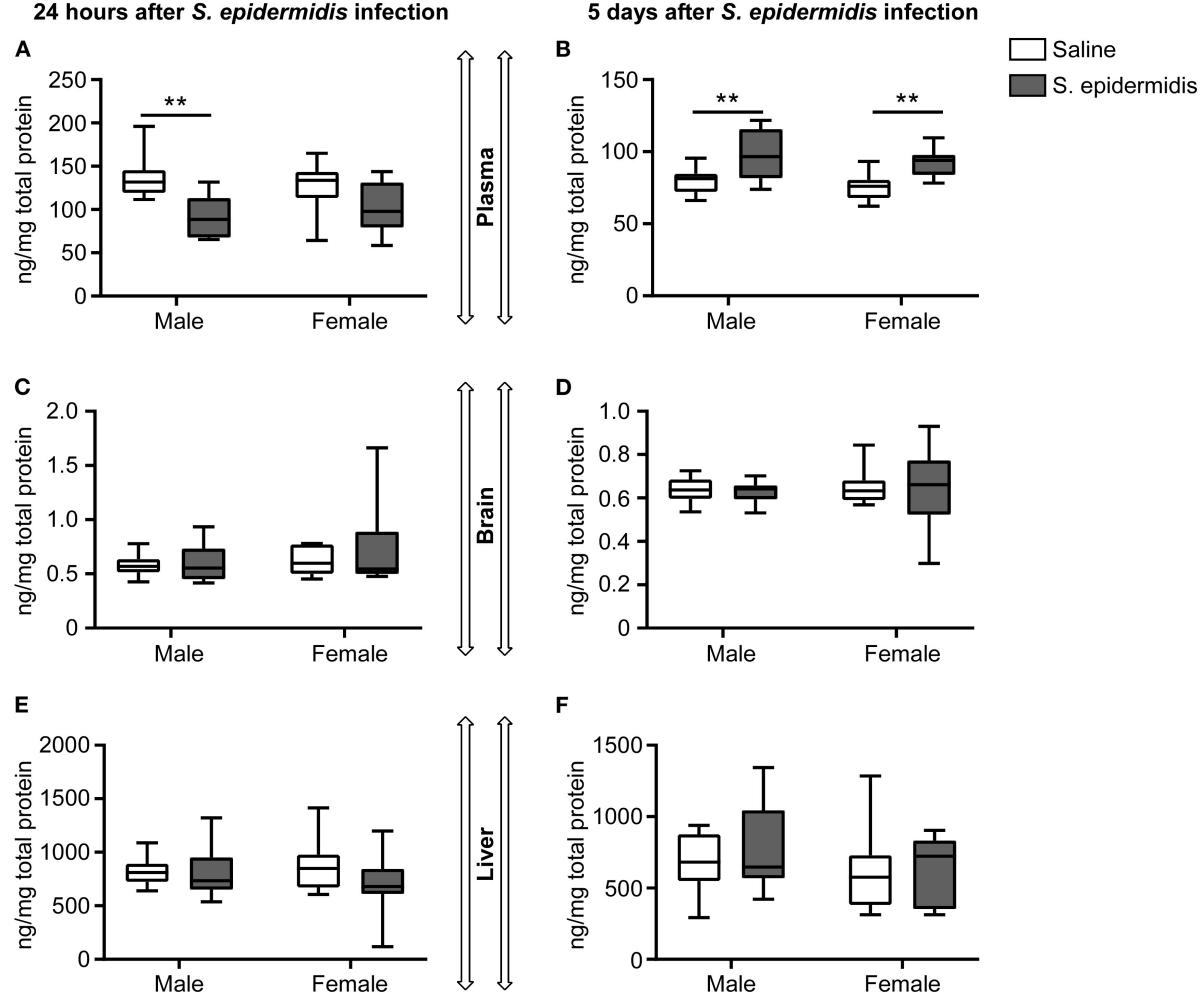
Plasma concentrations of C5a is reduced 24 h following *S. epidermidis* infection. PND4 mice were injected with saline or *S. epidermidis* and analyzed for C5a levels in the plasma, brain, and liver at **(A,C,E)** 24 h or **(B,D,F)** 5 days post infection (*n* = 10 mice/sex/group). Data are presented as median and 10–90^th^ percentile. Statistical comparison between the *S. epidermidis* and saline groups was done using Two-way ANOVA followed by *post-hoc* Sidak's multiple comparison test; **p < 0.01.

To study the effect of *S. epidermidis* infection on complement protein activation, we measured C5a levels in the plasma, brain, and liver by ELISA. Two-way ANOVA revealed no significant interaction between sex and bacterial infection at 24 h and 5 d after infection; but there was a significant main effect of bacterial infection on C5a levels in the plasma at both time points [*F*_(1, 36)_ = 16.53, *p* = 0.0002; *F*_(1, 36)_ = 21.71, *p* < 0.0001, respectively, Figures 7A,B]. Specifically, plasma C5a levels were significantly decreased 24 h after *S. epidermidis* injection in males compared to saline treated controls (*p* = 0.001). This difference was not observed in females (*p* = 0.09, Figure 7A). In contrast, there was an increase in the plasma level of C5a in both sexes 5 d after *S. epidermidis* injection (*p* = 0.002 in males and *p* = 0.007 in females, Figure 7B). No difference in C5a levels were found in the brain (Figures 7C,D) or in the liver (Figures 7E,F) in either sex or time point.

### *S. epidermidis* Alters C5 Signaling in the Liver

**Figure 8:**
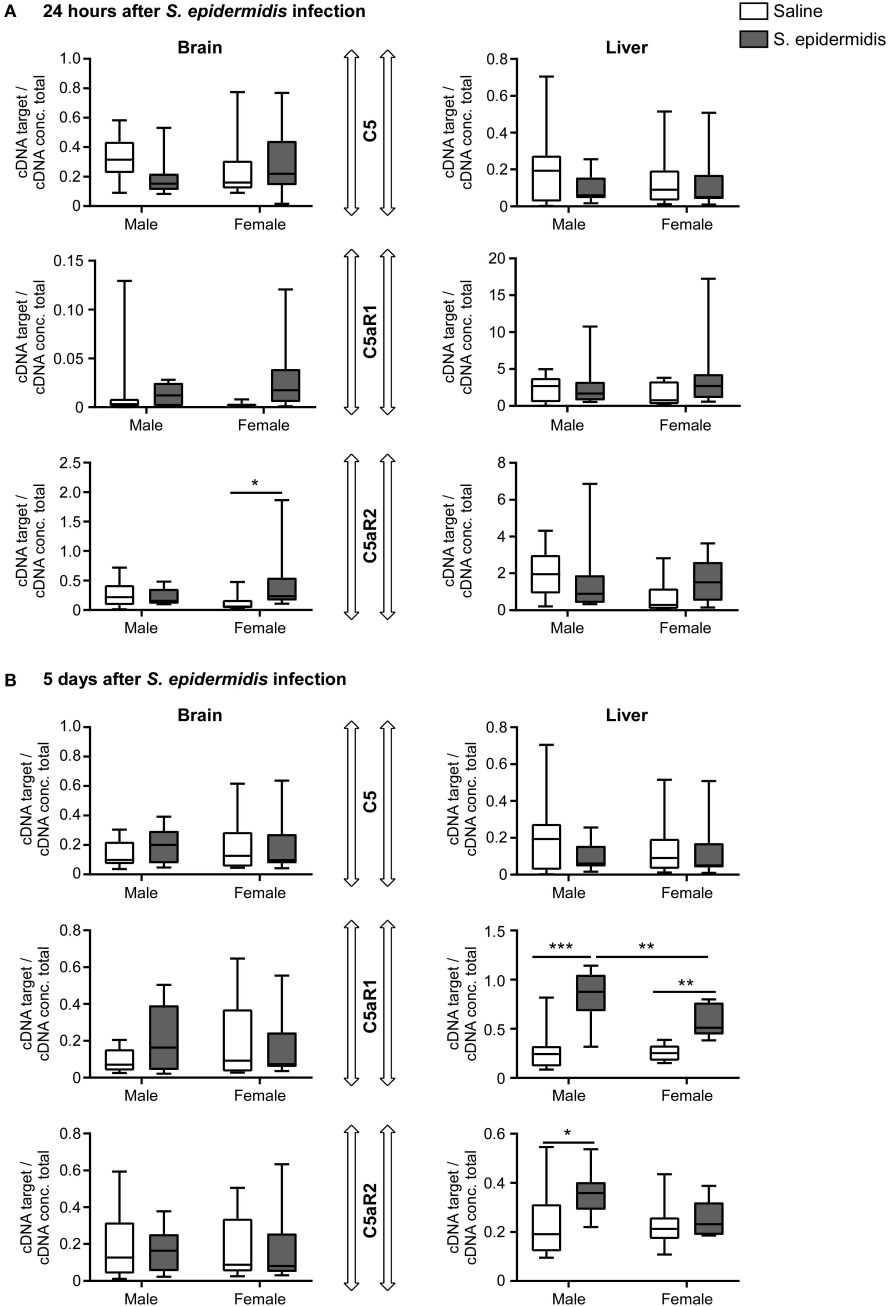
Complement component C5 and receptor expression following *S. epidermidis* infection. PND4 mice were injected with saline or *S. epidermidis* and mRNA involved in C5 signaling (C5, C5aR1, C5aR2) was analyzed by PCR at **(A)** 24 h or **(B)** 5 days in the brain and liver (*n* = 10 mice/sex/group). Data are presented as median and 10–90^th^ percentile. Statistical comparison between the *S. epidermidis* and saline groups was done using Two-way ANOVA followed by Sidak's multiple comparison *post-hoc* test; *p < 0.05, **p < 0.01, and ***p < 0.001.

To evaluate C5 signaling after *S. epidermidis* infection, we measured the mRNA expression of C5, C5aR1, and C5aR2 in the brain and liver. Analysis of C5, C5aR1, and C5aR2 expression in the brain and liver at 24 h and 5 d post infection revealed no significant interaction between sex and bacterial infection, but there was a significant effect of bacterial infection on the expression of C5aR1 and C5aR2 in the liver 5 d post infection [*F*_(1, 36)_ = 49.11, *p* < 0.0001; *F*_(1, 36)_ = 5.25, *p* = 0.02, respectively] and an effect of sex on the expression of C5aR1 in liver 5 d post infection [*F*_(1, 36)_ = 5.55, *p* = 0.02] (Figure 8). At 5 d after the infection, C5aR2 was only significantly upregulated in the liver of infected males compared to the saline group (*p* = 0.02), while C5aR1 expression in the liver was consistently upregulated in both sexes (*p* < 0.0001 in males and *p* = 0.002 in females; Figure 8B). The expression of C5aR2 was upregulated in the brain of infected female (*p* = 0.04) but not in male mice 24 h after *S. epidermidis* infection (Figure 8A).

## Discussion

To study the effects of perinatal infection on cerebral vulnerability, we examined the impact of *S. epidermidis*, the most common nosocomial infection in preterm infants, on HI injury in neonatal mice. Here, we show for the first time that the vulnerability to HI brain injury subsequent to *S. epidermidis* infection is both time and sex-dependent, with an increased sensitivity in males.

We have previously reported that *S. epidermidis* infection 14 h prior to HI in neonatal (PND4) mice sensitizes the brain to cerebral injury (14) and that intravenous injection of *S. epidermidis* at PND0 impairs brain development (26). *S. epidermidis* is known to activate TLR2 (27), but *S. epidermidis* induction of neonatal brain injury is both TLR2 dependent and independent (26). Furthermore, innate immune responses to *S. epidermidis* in preterm infants is known to be age-dependent (28). We and others have demonstrated that administration of specific TLR2 agonists prior to HI increases vulnerability to brain injury (12, 13). Previous studies have also demonstrated that vulnerability to HI following LPS-induced inflammation is dependent on the time interval between LPS and HI (15). This suggests that timing of the infection is important. Supporting this, we find in the present study that the time interval between *S. epidermidis* infection and HI induction determines the neuropathological outcome, at least in male mice.

Brain development is a sexually dimorphic process, including neurotransmission and/or genetic and metabolic responses (29, 30). Furthermore, perinatal complications are dependent on sex. For example, a higher incidence of preterm birth is observed in males (31) and HI affects male more often than female infants (32). Increased male prevalence has also been observed in several neurodevelopmental disorders such as autism (33) and attention deficit hyperactivity disorder (34, 35). Significant evidence points to sexual dimorphism of the inflammatory response being an underlying factor for the sexual bias in neuro-psychiatric disorders (25). An elegant experiment by Villa et al. recently demonstrated that female and male microglia are different and that female microglia transplanted into the brain of male mice maintain their sex-specific features and provide neuroprotection in males following adult stroke (36). Supporting previous evidence demonstrating increased male vulnerability to inflammation and brain injury, our present study shows that *S. epidermidis* selectively sensitizes males to HI brain injury.

In the present study, increased brain vulnerability in males was only observed when HI was induced at 24 h, but not 5 days post infection. To investigate the role of inflammation at these two time points, when sensitization was and was not identified, we performed cytokine and chemokine assays in the blood and brain of male and female mice. Although we observed a significant upregulation of several pro- and anti-inflammatory cytokines in the blood 24 h post *S. epidermidis* infection, only IL-2, CCL5, and IL-17 were regulated in a sex-dependent manner. At 5 d after infection, most cytokines had tapered back to baseline levels and only IL-6 and G-CSF remained upregulated in males. Consistent with our findings, PND10 rats of both sexes challenged with either a mix of bacterial or viral components showed an upregulation of blood (serum) cytokines and chemokines at PND12, returning to constitutive levels 5 d later (37).

There is increasing evidence supporting sex dimorphism in the cytokine response to certain bacterial infections, such as an overproduction of IL-2 in males (38). However, to our knowledge, there are no studies investigating sex-dependent differences of IL-2, CCL5, or IL-17 in neonatal sepsis. Clinical studies are conflicting and demonstrate either decreased (39) or increased CCL5 levels in the blood during neonatal sepsis (40). IL-17 is important for immune surveillance and reduced levels of IL-17A in human preterm neonates is associated with increased risk of bacteraemia (41). On the other hand, pathologically elevated IL-17 can be harmful in the neonate as it is also associated with inflammatory diseases such as asthma (42).

IL-6 and G-CSF have predictive value in neonatal sepsis (43, 44) and IL-6 remains high in newborns with poor outcome following hypoxic-ischemic encephalopathy (45). In this study, plasma IL-6 and G-CSF were upregulated in both sexes at 24 h, but stayed elevated at 5 days only in males. Similarly, in the brain, CCL2 was upregulated in both males and females at 24 h but only in males at 5 d after the infection. Thus, these cytokines and chemokines may be important factors that are more pronounced in males. Interestingly, in line with our previous findings, there was no clear translation of CCL2 concentration in the blood to expression in the brain (46).

Complement proteins and their proteolytic fragments are important for phagocytosis by neutrophils and monocytes (47) and deficiency of proteins in the complement cascade is associated with increased susceptibility to perinatal infection, mortality and low birthweight (48). We found a biphasic C5a response in the plasma of male mice following *S. epidermidis* infection, with reduced C5a levels 24 h later and an increase in C5a levels 5 d after infection. A similar trend was seen in females at 24 h and we cannot exclude that females may have had an initial significant decrease in C5a levels at other time points from those we evaluated in the present study. The biphasic pattern of C5a is similar to patients admitted to hospitals with different types of trauma leading to systemic inflammatory response syndrome where the downstream complement membrane attack complex (MAC) is decreased at day 1 and increased on days 2–7 (49). The initial decrease in C5a levels could be due to temporary complement depletion in the presence of the bacteria or inflammation. In addition, the production of extracellular proteases from *S. epidermidis* can inactivate human plasma serine and cysteine proteinase inhibitors and thereby degrade C5 (50, 51), which may also explain the early decrease in plasma C5a we observed. Consistent with this hypothesis, we found a reversal, or even an increase in plasma C5a levels 5 days after infection, a time when the bacterial load is significantly reduced in the blood (14).

The decrease in C5a levels may lead to worsened disease outcome as C5-deficient mice demonstrate impaired bacterial clearance (52) and a study of adult patients with sepsis-induced brain dysfunction revealed a correlation between decreased C5a levels with increased mortality (53). Neonatal rats demonstrate elevated expression of C5a and C5 receptors in the blood and brain following HI, which is reduced with therapeutic hypothermia, suggesting that C5a has an injurious effect in cerebral tissue (18, 19). In contrast, even though C5 receptor expression was increased in the liver, we did not find any major changes in the mRNA expression of C5 or C5 receptors in the brain of infected mice prior to HI. Future studies examining C5 signaling following the combination of *S. epidermidis* infection and HI may reveal stronger evidence for a role of C5 complement in brain injury. Furthermore, as C5 receptors are mainly expressed in microglia, future studies that examine cellular expression of C5 receptors upon *S. epidermidis* infection may provide additional supporting data.

In conclusion, our results demonstrate that neonatal hypoxia-ischemia added to an ongoing *S. epidermidis* infection in combination with enhanced inflammation increases the vulnerability of the developing brain in a time- and sex-specific manner. Increased expression of a number of cytokines and chemokines and decreased C5a protein in the blood were associated with increased vulnerability in males. These findings are consistent with a higher incidence of perinatal brain injury in newborn males (32). Overall, we provide fresh insight into how systemic *S. epidermidis* infection affects the developing brain.

## Data Availability Statement

All datasets generated for this study are included in the article/Supplementary Material.

## Ethics Statement

The animal study was reviewed and approved by Gothenburg Animal Ethical Committee (No. 663/2017).

## Author Contributions

This study was conceived and designed and the manuscript drafted by GG, JL, and CM. Experiments were performed by GG, PS, JL, and MA. Data analysis was performed by GG, JL, MA, and CE. All authors critically reviewed and edited the work.

## Conflict of Interest

The authors declare that the research was conducted in the absence of any commercial or financial relationships that could be construed as a potential conflict of interest.
