# Supplementary material for: Staphylococcus epidermidis Sensitizes Perinatal Hypoxic-Ischemic Brain Injury in Male but Not Female Mice
Source: Front Immunol. 2020 Apr 21;11:516. doi: 10.3389/fimmu.2020.00516 (PMC7186320; doi:10.3389/fimmu.2020.00516)
Supplement: Supplementary file 1 [file Image_1.pdf]

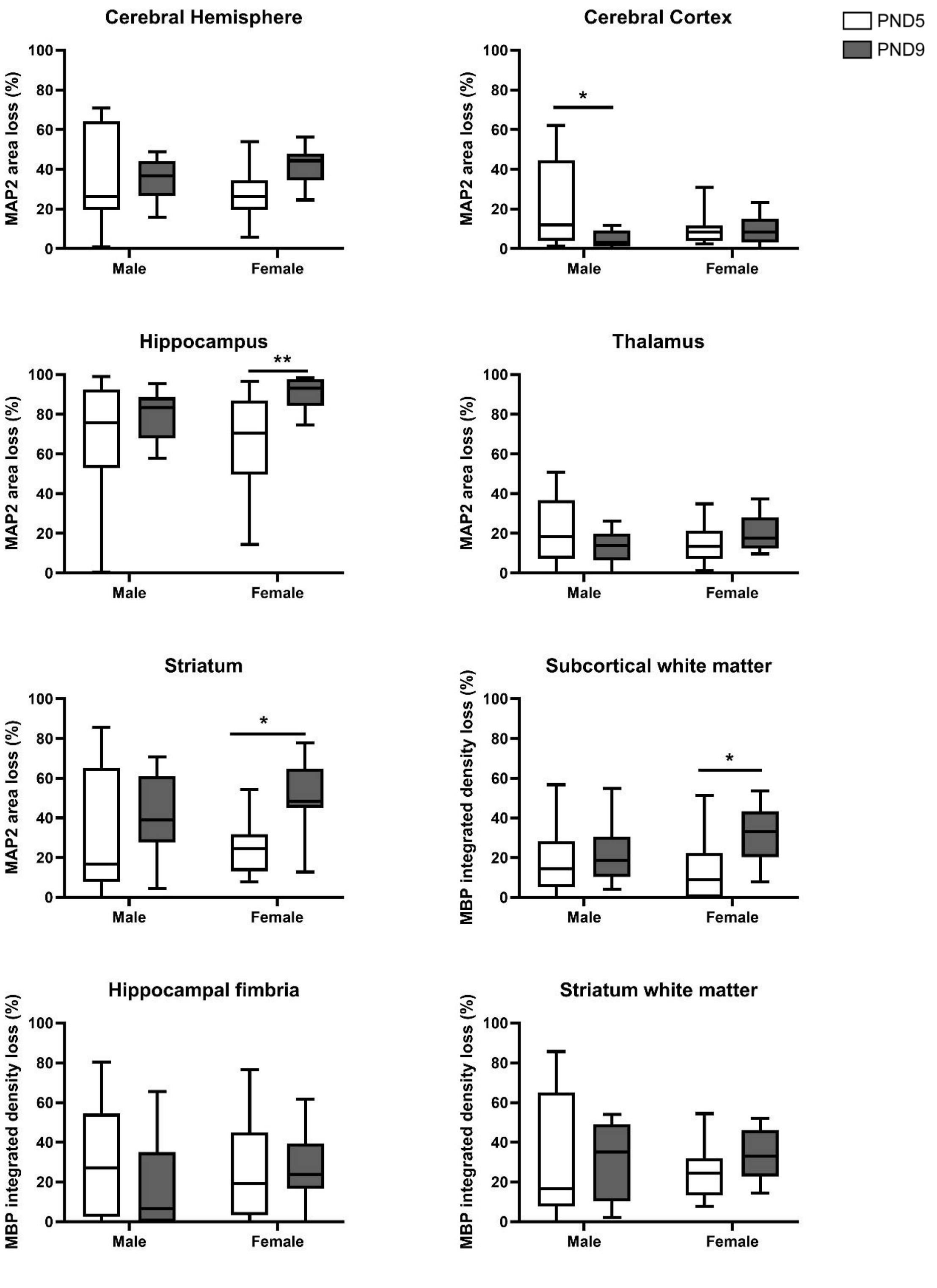

SUPPLEMENTARY FIGURE 1. Brain injury in mice subjected to hypoxia-ischemia. Mice were injected with saline at PND4, then subjected to 60 min of hypoxia (10% O_2) 24 h later (at PND5), or 50 min of hypoxia 5 d later (at PND9). At PND14-16, gray matter brain injury was assessed by microtubule-associated protein 2 (MAP-2) immunohistochemistry, while white matter injury was assessed by myelin basic protein (MBP) immunohistochemistry (n = 12 PND5 males; n = 17 PND5 females; n = 12 PND9 males, and n = 16 PND9 females). Data are presented as median and 10th – 90th percentile. Statistical comparison was performed between the PND5 and PND9 groups for each sex using Two-way ANOVA with Sidak´s multiple comparison *post-hoc* test; *p<0.05, and **p<0.01.
